# ^68^Ga-DOTATATE PET/CT in the Initial Staging of Well-Differentiated Gastroenteropancreatic and Non-Gastroenteropancreatic Neuroendocrine Tumors: Results of a Prospective Registry

**DOI:** 10.3390/cancers17030434

**Published:** 2025-01-27

**Authors:** Ur Metser, Roshini Kulanthaivelu, Julia Duder, Ricarda Hinzpeter, Simron Singh, Rebecca Wong, Sten Myrehaug, Daryl Gray, Patrick Veit-Haibach, Amit Singnurkar, Xuan Li, Shereen Ezzat

**Affiliations:** 1 University Medical Imaging Toronto, University Health Network, Mount Sinai Hospital & Women’s College Hospital, 610 University Ave, Suite 3-920, Toronto, ON M5G 2M9, Canadajuliaduder@gmail.com (J.D.); patrick.veit-haibach@uhn.ca (P.V.-H.); 2 Department of Medical Imaging, University of Toronto, Toronto, ON M5T 1W7, Canada; amit.singnurkar@sunnybrook.ca; 3 Odette Cancer Centre, Sunnybrook Health Sciences Centre, 2075 Bayview Avenue, Toronto, ON M4N 3M5, Canada; 4 Division of Medical Oncology, Department of Medicine, University of Toronto, Toronto, ON M5T 1P5, Canada; 5 Radiation Medicine Program, Princess Margaret Cancer Centre, 610 University Ave, Toronto, ON M5G 2M9, Canada; 6 Radiation Oncology, University of Toronto, Toronto, ON M5T 1P5, Canada; 7 Department of Radiation Oncology, Odette Cancer Centre, Sunnybrook Health Sciences Centre, 2075 Bayview Avenue, Toronto, ON M4N 3M5, Canada; sten.myrehaug@sunnybrook.ca; 8 Department of Surgery, London Health Sciences Centre—Victoria Hospital, Western University, 800 Commissioners Road East, London, ON N6A 3K7, Canada; daryl.gray@lhsc.on.ca; 9 Department of Medical Imaging, Sunnybrook Health Sciences Center, 2075 Bayview Avenue, Toronto, ON M4N 3M5, Canada; 10 Department of Biostatistics, University Health Network, 700 University Ave, Toronto, ON M5G 1X6, Canada; xuan.li@uhn.ca; 11 Department of Medicine, University of Toronto, Toronto, ON M5S 3H2, Canada; shereen.ezzat@uhn.ca; 12 Princess Margaret Cancer Centre, 610 University Ave, Toronto, ON M5G 2M9, Canada

**Keywords:** ^68^Ga-DOTATATE, PET/CT, neuroendocrine tumors, initial staging, stage

## Abstract

**Background and Objectives:** At diagnosis, the initial staging of well-differentiated neuroendocrine tumors (WD NETs) aids in treatment planning. The somatostatin receptor (SSTR)-PET has been recommended for staging of WD NETs although limited data are available on its impact on non-gastroeneteropancreatic (GEP) NETs. The main purpose of this study was to compare the stage migration after the addition of SSTR-PET to the workup of patients at the initial staging of GEP NETs to those with non-GEP NETs, and its potential impact on patient management. **Methods:** This prospective study included patients with WD NETs at initial staging. Demographic data, results of conventional and SSTR-PET staging, and SUVmax were recorded. Three panels of experts assessed the potential impact of SSTR-PET to management. **Results:** There were 482 patients, including 376 with gastroenteropancreatic (GEP) NETs and 106 non-GEP NETs with a median SUVmax of 34.7 [Q1, Q3: 22.8, 59.1]) and 19.0 [Q1, Q3: 7.9, 39.8]), respectively; *p* < 0.001. The discordant M-stage was recorded in 111/473 patients (23.5%). PET suggested a higher stage in 78/369 GEP NETs (21.1%), including the detection of extrahepatic metastatic disease in 42/114 (36.8%) patients with liver metastases only on CI. For non-GEP NETs, PET suggested a higher stage in 10/104 (9.6%) and CI suggested a higher stage in 15/104 (14.4%), with CI detecting liver metastases more frequently. The potential impact to management for patients with discordant M-stage was scored as moderate to high between 57/101 (56.4%) and 79/101 (78.2%) of patients. **Conclusions:** One in five patients are upstaged following SSTR-PET, more frequently with GEP NETs than others. SSTR-PET identifies extrahepatic metastatic disease in >1/3 of patients with presumed liver-only metastases on CI. Stage migration following SSTR-PET may result in frequent moderate or significant management change.

## Simple Summary

The disease extent of newly diagnosed well-differentiated neuroendocrine tumors determines patient management. Staging is routinely performed using anatomical imaging (CT and/or MRI). The purpose of the current prospective study was to determine the impact of adding the somatostatin receptor (SSTR)-PET on disease stage and whether stage migration following PET may impact patient management. We confirmed upstaging following SSTR-PET in one in five patients, more frequently with gastroenteropancreatic neuroendocrine tumors than other histologies. This may result in a moderate to high impact to the therapy plan in more than half of the upstaged patients.

## 1. Introduction

Neuroendocrine tumors (NETs) are an uncommon heterogeneous group of neoplasms that arise from cells of the endocrine and nervous systems. The most common subtypes are bronchopulmonary, followed by gastroenteropancreatic NETs (GEP NETs). The latter may arise in the stomach, small bowel, large bowel, or pancreas and those of unknown origin. Less common sites of primary tumors include medullary thyroid cancer, thymic neuroendocrine tumors, and pheochromocytoma/paragangliomas [1,2,3]. NETs can be functioning, with hormone hypersecretion which often produces symptoms. However, approximately 60% are non-functioning, with non-specific clinical presentation and delayed diagnosis [2]. In recent years, somatostatin receptor PET (SSTR-PET), mostly using gallium-68 (^68^Ga)-labeled somatostatin analogues (^68^Ga-DOTA-TOC, ^68^Ga-DOTA-TATE, and ^68^Ga-DOTA-NOC) have been incorporated clinically for the identification and staging of NETs. Appropriate use criteria (AUC) for SSTR-PET imaging in NETs were developed by a panel of experts including representatives from the Society of Nuclear Medicine and Molecular Imaging, European Association of Nuclear Medicine, American Society of Clinical Oncology and the North American Neuroendocrine Tumor Society, and last updated in 2020 [4,5]. They evaluated the appropriateness of SSTR-PET in 12 potential clinical scenarios, scoring each scenario on a 9-point scale with scores 7–9 indicating appropriate and 4–6 indicating possibly appropriate for the specific indication. There were three scenarios for the initial staging of histologically proven NETs including the initial staging after histologic diagnosis of NETs, localization of primary tumor in patients with known metastatic disease but unknown primary site, and staging of NETs before planned surgery, for which appropriate use criteria scores of 9, 9, and 8 were assigned, respectively. These appropriate use criteria are limited to GEP NETs due to the lack of comparable evidence for the use of SSTR-PET in other disease subtypes.

^68^Ga-DOTATATE (NETSPOT^®^) was approved by the FDA for clinical use in the United States in June 2016, while in Canada, ^68^Ga-DOTATATE was approved for clinical use by Health Canada 4 years later. To provide access to patients prior to receiving regulatory approvals and to obtain larger scale data on the utility of the test, Ontario-Health Cancer Care Ontario launched the PET NET Registry (NCT03873870). The PET NET registry assessed the diagnostic performance of ^68^Ga-DOTATATE PET/CT for various clinical scenarios, including the initial staging of NETs. The primary aim of the current analysis was to determine the frequency of stage migration after the addition of SSTR-PET to the workup of patients with a histologically diagnosed well-differentiated NET at initial presentation. Secondary aims included the detection rate of a primary tumor site in patients with metastatic NET of unknown primary after conventional workup, comparison of stage migration after SSTR-PET to conventional workup for GEP NETs versus non-GEP NETs, and to assess the potential impact of stage migration on patient management.

## 2. Methods

The PET NET registry (NCT03873870) was a prospective Ontario Cancer Research Ethics Board approved registry (OCREB ID#1725). Written informed consent was obtained from all participants. The current analysis focused on consecutive patients referred for the initial SSTR-PET staging of an NET from 11 different centers across the province of Ontario, Canada over a 3-year period (1 April 2019 and 31 March 2022). Eligible patients included patients with a histological diagnosis of a well-differentiated neuroendocrine tumor, with or without a known primary tumor site. All patients underwent conventional workup (CT ± MR) as per the standard of care at the discretion of the treating oncologist, prior to inclusion in the registry. CI exams were interpreted locally by an experienced radiologist with available clinical information including histological diagnosis.

### 2.1. PET/CT Protocol and Image Interpretation

PET/CT was performed at one academic hospital. PET acquisition started 60 min (range: 50–90) after the injection of 100–200 MBq (2.7–5.4 mCi) of ^68^Ga-DOTATATE. During uptake time, a water-soluble oral contrast was given for bowel opacification on CT. Patients were positioned supine with their arms outside of the field of view. PET/CT was performed on a Biograph mCT 40 scanner (Siemens Healthineers, Erlangen, Germany). A low-dose CT without intravenous contrast was used for attenuation correction as per standard departmental protocols. Overall, 5–9 bed positions were obtained as per patient height (2–5 min/bed position). ^68^Ga-DOTATATE PET/CT was interpreted on a dedicated workstation (Thinking Systems PACSCloud, St. Petersburg, FL, USA) by one of 6 readers with 9–22 years of experience interpreting PET scans (median, 15 years). Image interpretation criteria were in line with the European association of Nuclear Medicine guidelines [6].

### 2.2. Data Abstraction and Management Plan

Demographic data including age and gender, location of primary tumor, tumor grade, site of biopsy (primary tumor and/or metastases), disease extent including locoregional and distant metastases as suggested on conventional workup (CT ± MR) prior to PET and ^68^Ga-DOTATATE PET/CT, were tabulated. For patients with a metastatic NET of an unknown primary tumor site, the detection of a primary site on PET was recorded. For patients with GEP NETs, the N and M stage as per the 8th edition of the AJCC cancer staging manual was recorded. As there are different staging algorithms for non-GEP NETs depending on the site of primary tumor, for these patients, the absence or presence of locoregional nodal metastases and distant metastases (N0/N1 and M0/M1) and the site and extent of distant metastases were tabulated. For all patients with distant metastases, the distribution and extent of metastatic disease were recorded. For each patient, the highest SUVmax and Krenning score assigned were tabulated.

Finally, the pre-PET treatment plan was recorded by the referring physician considering all available clinical information, conventional imaging workup and when available, multidisciplinary tumor board review. To assess the impact of stage migration following PET on patient management, the recorded pre-PET patient management plan was assessed after PET for all patients with discordant M-stage by 3 independent teams of raters from 3 disciplines (endocrine oncology, radiation oncology, and molecular imaging). The raters had access to clinical and imaging data as well as pre-PET planned management. The 3 teams assessed the potential impact of ^68^Ga-DOTATATE PET/CT on clinical management in these cases using the following scale (adapted from Ng et al. [7]):

N. No/unknown impact:

N0 = no impact; PET findings are unlikely to impact the pre-PET management plan;

N1 = no SSTR-2 overexpressing disease on PET; uncertain impact.

A. Low impact. For instance, the detection of additional metastases in a patient already planned for systemic therapy. PET may have prognostic implications but unlikely to change management.
B. Moderate impact. For instance, the detection of limited additional metastases in patients being considered for debulking surgery or for liver-directed therapy, in which a therapy plan may be amended.
C. High impact. For instance, the detection of metastases in patients with no metastases on conventional imaging; or detection of soft tissue and/or widespread bony disease in addition to other known diseases in patients planned for surgery (resection of primary tumor ± debulking of metastatic disease); or detection of new liver lobe and/or extrahepatic disease in patients undergoing liver-directed therapy (such as embolization), or surgical liver debulking.

### 2.3. Statistical Analysis

Descriptive statistics are provided for the characteristics of patients. Frequency (percentage) is provided for categorical variables such as gender, tumor grade, and primary tumor site. The mean (SD), median (Q1, Q3), and range (min, max) are summarized for continuous variables such as age, SUVmax, and Krenning score. Wilcoxon Rank-Sum test is applied to compare SUVmax and Krenning scores between GEP NET patients and non-GEP NET patients. Kruskal–Wallis test is used to compare SUVmax among tumor grades. Chi-square test or Fisher’s exact test is employed to compare categorical variables between groups. Cohen’s Kappa tests are used to assess the agreement among raters. Kappa coefficient ≤0, 0.01–0.20, 0.21–0.40, 0.41–0.60, 0.61–0.81, and 0.81–1.00 indicate no, slight, fair, moderate, substantial and almost perfect agreement, respectively. The statistical significance level is 0.05. The statistical analyses were performed using R 4.3.1.

## 3. Results

### 3.1. Demographics

There were 482 patients referred for initial staging of a histologically proven well-differentiated NET including 246 men (51%) and 236 women (40%), with a mean age (±SD) of 60.7 ± 13.8 years (range 17, 90). Primary tumor sites in the 376 patients with a GEP NET included the stomach (*n* = 24; 6.4%), small bowel (*n* = 187; 49.7%), colorectal (*n* = 35; *n* = 9.3%), appendix (*n* = 27; 7.2%), and pancreas (*n* = 103; 27.3%). There were 106 patients with non-GEP NETs with primary tumor sites in lung (47/106; 44.3%), unknown primary site (34/106; 32.1%), pheochromocytoma/paraganglioma (*n* = 11; 10.4%), other primary sites (*n* = 14; 13.2%). Tumor grades were G1 in 258/482 (54%), G2 in 138/482 (29%), G3 in 27/482 (6%), and unknown in 59/482 (12%). The pre-PET workup included MR in 115/482 (23.9%) of patients.

### 3.2. SUVmax and Krenning Score

There was a higher median SUVmax recorded for GEP NET patients (34.7 [Q1, Q3: 22.8, 59.1]) than for non-GEP NET patients (19.0 [Q1, Q3: 7.9, 39.8]); *p* < 0.001. The median (Q1, Q3) of the Krenning scores recorded for GEP NETs and non-GEP NETs were 4 (4,4) and 3 (2,4), respectively; *p* < 0.001. However, there was no significant difference in the median SUVmax [Q1, Q3] for patients across the various tumor grades: G1 (34.0 [Q1, Q3: 18.7, 58.3), G2 (30.0 [Q1, Q3: 16.4, 50.9], G3 (33.4 [Q1, Q3: 16.8, 51.2], or unknown grade (28.0 [Q1, Q3: 16.2, 47.2]; *p* = 0.59.

### 3.3. Detection of Primary Tumor

**Figure 1:**
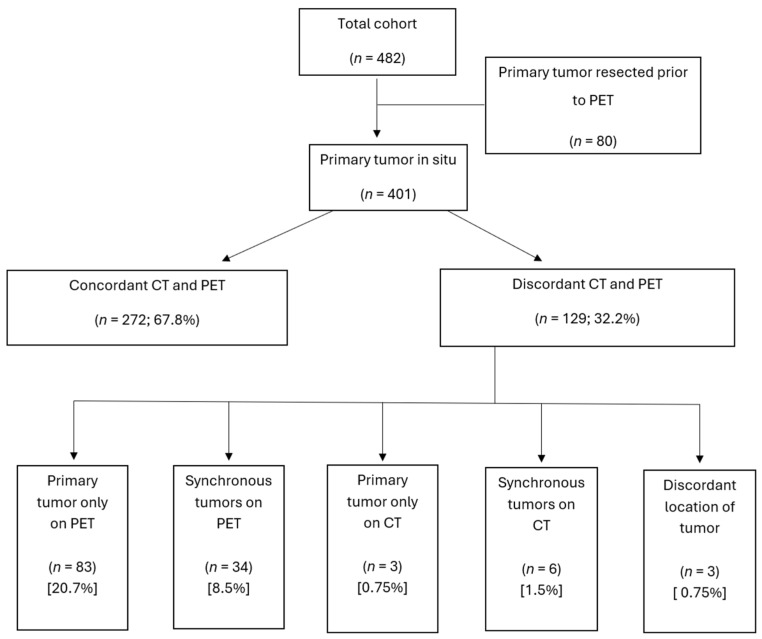
Detection of primary tumor on conventional imaging and PET.

There were 81 patients whose primary tumor was resected prior to PET (Figure 1). PET and CI were concordant for the site of primary tumor in 272/401 patients (67.8%). PET suggested primary tumor sites not seen on CI in 117/401 patients (29.2%), including 83 patients in whom a primary tumor was not identified on CI, and 34 additional patients in whom synchronous primary tumors (such as multifocal small bowel tumors) were identified on PET. Of patients with metastatic NET of an unknown primary tumor site, 11/34 (32.3%) had primary tumors suggested on SSTR-PET.

### 3.4. N Stage

There were 473 patients (368 with GEP NETs and 105 with non-GEP NETs) whose regional nodal status could be evaluated on PET and CI (missing data/not evaluable in 9), with 157/473 (33.2%) who were node positive on CI and 199/473 (42.1%) who were node positive on PET; *p* < 0.001 (Table 1). For patients with GEP NETs, CI was positive in 126/368 (34.2%) and PET was positive in 165/368 (44.8%). For those with non-GEP NETs, CI was positive in 32/105 (30.5%) and PET was positive in 35/105 (33.3%).

### 3.5. M Stage

There were 473 patients (369 with GEP NETs and 104 with non-GEP NETs) who had complete imaging data for the evaluation of distant metastases. The M stage for the entire cohort, and separately for those with GEP NETs and non-GEP NETs is presented in Table 2, Table 3 and Table 4. The distribution of extrahepatic metastases by modality is presented in Table 5.

**Figure 2:**
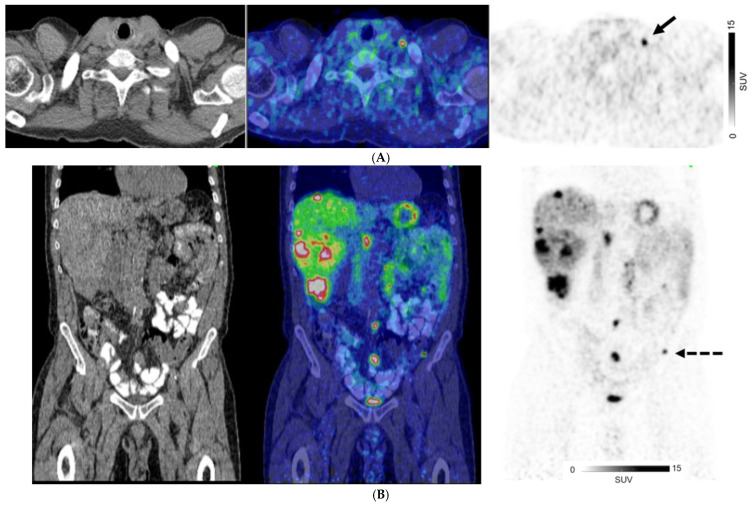

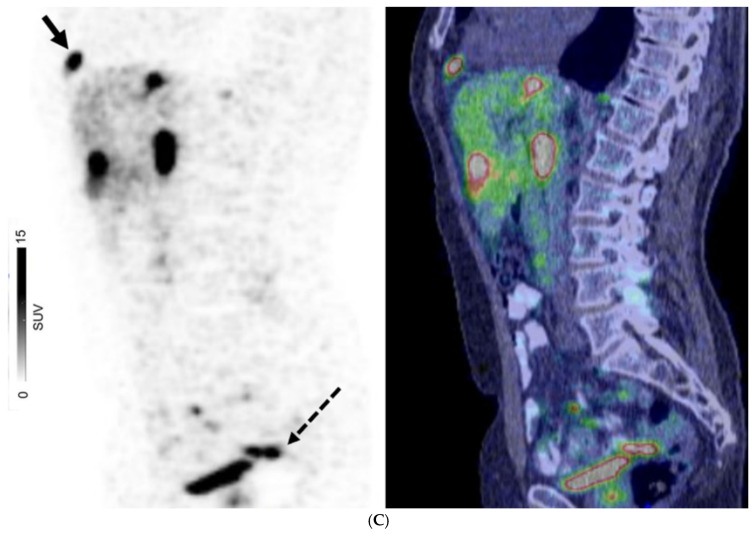
A 67-year-old man with well-differentiated G1 small bowel NET, Ki 67 = 3% metastatic to liver. Staging CT showed mid-ileal tumor, regional nodal metastases, and bilobar liver metastases [CT stage: n1M1a]. ^68^Ga-DOTATATE PET/CT (**A**,**B**) Axial and Coronal—CT (**left**), fused PET/CT (**middle**) and PET (**right**); (**C**) Sagittal—Fused PET/CT (**left**) and PET (**right**) shows in addition, extrahepatic metastases including extraregional nodes including supraclavicular and cardiophrenic nodes (arrows in (**A**,**C**)), and peritoneal metastases (dotted arrows in (**B**,**C**)); overall, PET stage: n1M1c.

**Figure 3:**
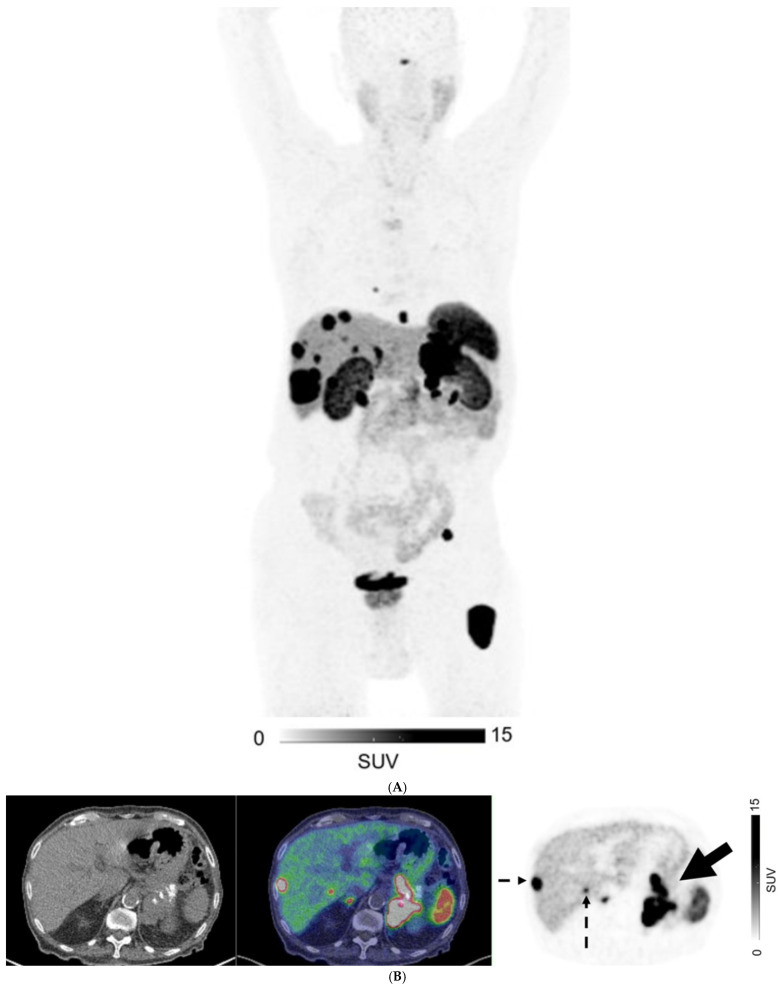

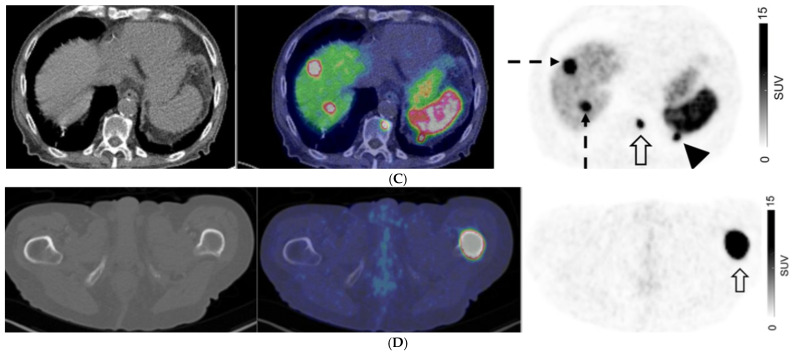
A 76-year-old man with well-differentiated G3 pancreatic neuroendocrine tumor (SUVmax 131.5, Krenning score 4), with bilobar liver metastases on CT. ^68^Ga-DOTATATE PET (**A**) MIP image; (**B**–**D**) Axial images: CT (**left**), fused PET/CT (**middle**), and PET (**right**). PET shows an intensely-avid locally advanced pancreatic tail mass (SUVmax = 132; solid arrow in (**B**)), liver metastases (biopsy proven; dotted arrows in (**B**,**C**)), peritoneal deposit (arrowhead in (**C**)) and bone metastases (open arrows in (**C**,**D**)).

For patients with GEP NETs, no distant metastases (M0) were observed on both CI and PET in 186/369 (50.4%) of patients. There were 83 patients with GEP NETs who had discordant M-stage on CI and PET, in which PET suggested a higher stage in 75/83 patients (90.4%) and lower stage in 8/83 (9.6%) (Figure 2). For those with GEP NETs, extrahepatic metastatic disease was identified by PET in 42/114 (36.8%) patients where CI showed only liver metastases (M1a) (Figure 3).

**Figure 4:**
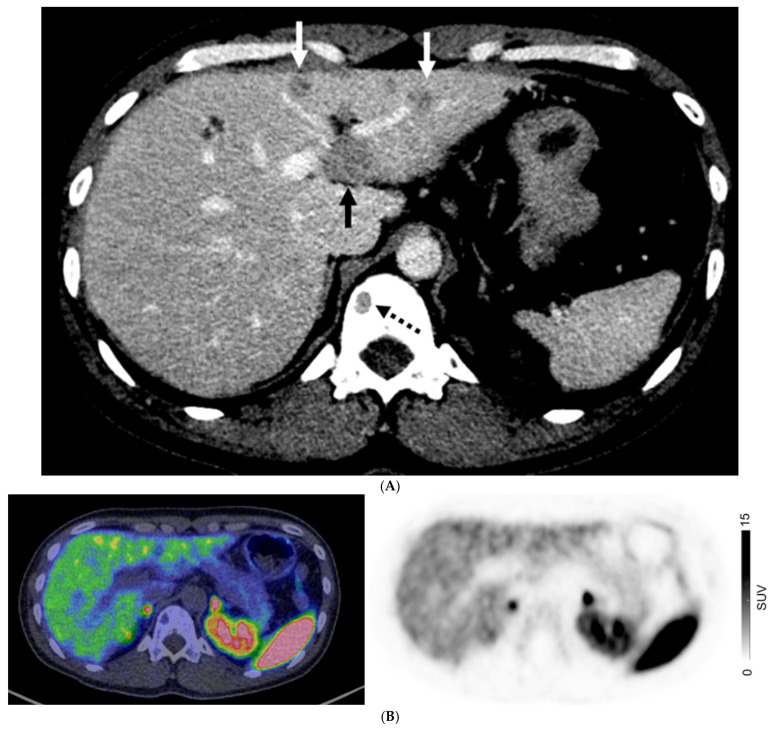
A 40-year-old man with metastatic well-differentiated G3 neuroendocrine tumor, unknown primary. Contrast-enhanced CT ((**A**) Axial image) shows multiple liver deposits (histologically proven; solid arrows) and lytic bone lesion at T12 vertebral body (dotted arrow). ^68^Ga-DOTATATE PET/CT ((**B**) Fused axial PET/CT image left and Axial PET image right) show no corresponding focal radiotracer uptake.

For patients with non-GEP NETs, M0 was confirmed on both CI and PET in 56/104 (53.9%) patients. There were 25/104 (24%) with discordant M-stage on CI and PET. CI and PET suggested liver metastases in 34/104 (32.7%) and 23/104 (22.1%) patients, respectively, and extrahepatic distant metastases in 23/104 (22.1%) and 25/104 (24%) patients, respectively. There were 13/48 patients (27.1%) with non-GEP NETs who had distant metastases on CI but not on PET; 10 of whom had histologically proven distant metastases (Figure 4). When assessing the 47 patients with lung NETs, only 5/47 patients (10.6%) had a higher stage on PET, all of whom had bone metastases with or without liver metastases, while 6/47 (12.8%) had a higher stage assigned on CI, with metastases seen in the liver, lungs, and ovaries.

### 3.6. Potential Impact on Management

The potential impact of SSTR-PET on the pre-PET management plan was assessed in 101 patients with discordant M-stage who had complete datasets. A moderate or high impact score was assigned to most cases, ranging from 57/101 (56.4%) to 79/101 (78.2%) across the three groups of reviewers (Table 6). Using the 5-point impact scoring algorithm, there was overall fair agreement amongst the three groups of reviewers (52.2% agreement; Kappa = 0.287). When grouping no-low vs. moderate-to-high impact, there was 75.9% agreement amongst the reviewers (Kappa = 0.379). There were 89/101 cases (88.1%) in which at least two groups of readers agreed on a specific impact score. For GEP NETs and non-GEP NETs patients, moderate-to-high scores were assigned to the impact of SSTR-PET on the pre-PET management plan between 51/83 (61.4%) to 71/83 (85.5%) and between 6/18 (33.3%) to 12/18 (66.7%) patients, respectively. When applying this across the entire study population, the potential impact on management would be between 57/473 (12.1%) and 79/473 (16.7%) of patients with well-differentiated NETs.

## 4. Discussion

The use of SSTR-PET in the initial staging of well-differentiated NETs resulted in upstaging of nearly one in five patients, including the identification of metastases in nearly 8% of in patients with no distant metastases on CI. Upstaging after PET was more prevalent for GEP NETs (21.1%) than for non-GEP NETs (8.7%) in whom CI suggested a more extensive disease in 13.5% of patients. This may reflect the less frequent overexpression of SSTR-2 in non-GEP NETs as compared to GEP NETs, as also supported by the significantly lower SUVmax and Krenning scores recorded in these patients. Specifically, in patients with lung NETs, who comprised the largest contribution to non-GEP cohort, nearly a quarter of patients had discordant M-stage on PET and CI with 60% of them showing a more extensive disease on CI. Similar findings were found in a prior retrospective study which showed that nearly 30% of patients with lung NETs had no or weak expression of SSTR on PET and only 44% had uniformly positive receptor expression [8]. This suggests that for non-GEP NETs, the addition of SSTR-PET to CI may be less impactful. The overall higher detection rate of distant metastases in the current study is in line with prior reports, including a prospective study on 131 patients with GEP NETs or NETs of an unknown primary site which reported a higher tumor detection rate (primary tumor and metastases) on SSTR-PET as compared to CT/MR (95.1% vs. 45.3%, respectively; *p* < 0.001) [9].

The management of patients with NETs is complex and depends on disease grade, stage, primary site (GEP vs. lung vs. other) and disease burden [10,11,12,13,14,15]. Long-term survival has been reported for patients undergoing complete primary tumor resection and metastasectomy, when feasible [16,17], highlighting the importance of identifying all disease sites prior to primary therapy. In the current study, PET identified a primary tumor when none was found on CI in more than 20% of patients and showed further synchronous tumors in an additional 9%. Accurate delineation of disease extent, including synchronous tumors, presence or absence of distant metastases, their location and extent are crucial for selecting and planning the optimal management for patients presenting with an NET. In patients with symptomatic liver metastases, various liver-directed therapies may be offered, including surgical debulking, hepatic arterial embolization, and percutaneous ablative procedures [18,19]. In select patients with non-resectable liver metastases and no extrahepatic metastatic disease, liver transplant may represent another therapeutic option, with a 10-year survival rate of 52% in one series [20]. Accordingly, an important observation from our data is that SSTR-PET detected extrahepatic metastatic disease in more than a third of GEP NET patients with presumably limited metastatic disease to the liver on CI (42/114; 36.8% of patients assigned stage M1a on CI). The detection of additional disease burden may impact future treatment selections such as peptide receptor radionuclide therapy vs. liver-directed therapy, including liver transplant. The role of CI in the surgical management of patients with NETs is well-documented. Contrast enhanced imaging is crucial in assessing the anatomic extent of tumor including vascular involvement or invasion of adjacent structures, which may impact the extent of surgery or mode of therapy. From our data and prior data [9], SSTR-PET identifies more lesions and more disease sites than CI and both imaging methods should be used to optimally plan patient management.

In the current study, the potential impact of SSTR-PET on management decisions was performed post hoc with three groups of raters. Although an interrater agreement in the current study was only fair, perhaps due to the use of a 5-point scoring scale, a moderate-to-high potential impact of PET to pre-PET therapy plan was noted in more than half of the patients who had stage discrepancy on CI and SSTR-PET. For the entire study population, staging data obtained from SSTR-PET performed for the initial staging of patients with well-differentiated NETs may have a moderate or high impact on the pre-PET planned management in 12.1–16.7% of patients, with a higher impact in GEP NET patients. This is likely a conservative estimate, as it may not account for the impact of SSTR-PET on the management of patients in whom a greater extent of disease is observed but which does not translate to a change in disease stage (for example, an additional site of extrahepatic metastases in a patient planned for debulking surgery). A prior study by Sadowski et al. documented a change in management recommendation in 32.8% of the cohort [9]. Furthermore, a prior systematic review and meta-analysis on the impact of SSTR-PET on the management of patients with NETs suggested a change in management in 44% of patients (range 16–71%), with intermodality change in management in nearly a quarter of patients in whom management change was documented [21].

To the best of our knowledge, this is the largest prospective study to date to report the contribution of SSTR-PET to the initial staging of well-differentiated NETs, including more than 20% with non-GEP NETs. Patients included were from multiple cancer centers across the province of Ontario, Canada. We were able to demonstrate a significant stage migration after SSTR-PET especially for those with GEP NETs, which may frequently impact management decisions. There are several limitations to the current study. First, there was heterogeneity in patient population prior to undergoing SSTR-PET (with primary tumors resected in 81 patients prior to SSTR-PET), and in the pre-PET workup with some patients receiving MR (liver, spine, or other) in addition to CT. This is consistent with the heterogeneity of the initial presentation of neuroendocrine tumors and the variable clinical workup associated with them. Some may present incidentally at the time of surgery with other clinical diagnoses, e.g., incidentally in an appendectomy specimen; while others may be symptomatic due to presentation with hormone hypersecretion or metastatic disease. The patient population in this study is a snapshot of patients presenting with an NET and referred for SSTR-PET over a 3-year period. Second, PET was interpreted by one of six experts locally, as would be seen clinically, rather than a central review. Third, the pre-PET management plan was determined by the referring expert physician and not uniformly by a specific study team. This may introduce variability in proposed management plans due to differences in levels of experience of the referring physician. However, the management plan was developed by the referring physician with all available clinical information, including multidisciplinary tumor board discussion, when available. Furthermore, we did not have data collected on the actual treatment received after PET and the potential impact to patient management was not assessed by the referring physician but rather by a post hoc expert panel. This evaluation was performed for patients in whom there was a discrepancy between stages on PET and CI, in whom PET may potentially impact management. Although an agreement between the groups of reviewers was only fair, a moderate to significant impact was assigned in up to one in six patients.

## 5. Conclusions

In conclusion, there is a high impact for SSTR-PET in the initial staging of patients with GEP NETs, with upstaging in >20% of patients, as compared to <10% for those with non-GEP NETs. SSTR-PET identifies extrahepatic metastatic disease in more than a third of patients with presumed liver-only metastases on CI and may help guide treatment decisions in patients being considered for liver-directed management. Stage migration following SSTR-PET may result in a moderate or significant management change in 12.1–16.7% of patients.

## Figures and Tables

**Table 1:** N stage on conventional imaging (CI) and PET.

		PET	
		N0	N1
CI	N0	251	65
	N1	23	134

N0 = regional node negative; N1 = regional node positive; *p* < 0.001. Blue fields indicate concordant stage for PET and CI.

**Table 2:** M stage for the entire cohort on conventional imaging (CI) and PET; * Fisher exact test, *p* < 0.001.

			PET *			
			M0	M1		
				Liver	EH	Both
CI *	M0		242	15	15	6
	M1	Liver	12	75	3	45
		EH	4	0	14	4
		Both	5	0	2	31

CI = conventional imaging; M0 = no distant metastases; M1 = distant metastases; Liver = metastases limited to liver; EH = extrahepatic metastases; Both = both hepatic and extrahepatic metastases. Blue fields indicate concordant stage for PET and CI.

**Table 3:** M stage for patients with gastroenteropancreatic neuroendocrine tumors (GEP NETs).

		PET			
		M0	M1a	M1b	M1c
CI	M0	186	14	14	5
	M1a	6	66	2	40
	M1b	2	0	6	3
	M1c	0	0	0	25

CI = conventional imaging; M0 = no distant metastases; M1a = metastases limited to liver; M1b = extrahepatic metastases; M1c = both hepatic and extrahepatic metastases. Blue fields indicate concordant stage for PET and CI.

**Table 4:** M stage for patients with non-gastroenteropancreatic neuroendocrine tumors (non-GEP NETs).

			PET			
			M0	M1		
				Liver	EH	Both
CI	M0		56	1	1	1
	M1	Liver	6	9	1	5
		EH	2	0	8	1
		Both	5	0	2	6

CI = conventional imaging; M0 = no distant metastases; M1 = distant metastases; Liver = metastases limited to liver; EH = extrahepatic metastases; Both = both hepatic and extrahepatic metastases. Blue fields indicate concordant stage for PET and CI.

**Table 5:** Distribution of extrahepatic metastases for the entire cohort by imaging modality.

Site of EHD	CI (*n* = 60)	PET (*n* = 120)
Bone	15 (3.2%)	67 (14.2%)
Extraregional LN	18 (3.8%)	43 (9.1%)
Peritoneum	13 (2.8%)	40 (8.5%)
Lung	16 (3.4%)	17 (3.6%)
Other	16 (3.4%)	25 (5.3%)

EHD = extrahepatic metastatic disease; CI = conventional imaging; *n* = denotes the number of patients with EHD for each modality; percentages denote the prevalence of each EHD site in relation to the entire sample (*n* = 473); note—the patient may have more than one site of metastatic disease.

**Table 6:** Impact score distribution for each reviewer group (R1-R3). N0 = no impact; N1= no over-expressing disease on SSTR-2. A/ B/ C = low, moderate, high impact, respectively.

Impact Score	R1	R2	R3
N0	1 (0.9%)	1 (0.9%)	0 (0%)
N1	11 (10.9%)	11 (10.9%)	5 (5%)
A	32 (31.7%)	16 (15.8%)	17 (16.8%)
B	14 (13.9%)	31 (30.7%)	28 (27.7%)
C	43 (42.6%)	42 (41.6%)	51 (50.5%)

## Data Availability

The data presented in this study are available on request from the corresponding author.
